# Health Data Science — A New Science Partner Journal Dedicated to Promoting Data for Better Health

**DOI:** 10.34133/2021/9843140

**Published:** 2021-06-09

**Authors:** Qimin Zhan

**Affiliations:** National Institute of Health Data Science at Peking University, China

Health data and cutting-edge technologies empower medicine and improve healthcare. It has become even more true during the COVID-19 pandemic. Through coronavirus data sharing and worldwide collaboration, the speed of vaccine development for COVID-19 is unprecedented. Digital and data technologies were quickly adopted during the pandemic, showing how those technologies can be harnessed to enhance public health and healthcare. A wide range of digital data sources are being utilized and visually presented to enhance the epidemiological surveillance of COVID-19. Digital contact tracing mobile apps have been adopted by many countries to control community transmission. Deep learning has been utilized to achieve various solutions for COVID-19 disruption, including outbreak prediction, virus spread tracking, diagnosis and treatment, vaccine discovery, and drug research.

Abundant health data has been generated from various sources, including electronic health records (EHRs), surveillance data, administrative and claim information, wearable medical devices, genomic sequencing, and research data [1]. Additionally, healthcare data can be combined with information from social media, occupational information, geographical location, and economic and environmental data to disentangle complex health challenges [2]. Cutting-edge technologies have important roles to play in extracting the value of health data. The application of blockchain technology provides the possibility of efficiently and securely sharing health data while protecting privacy. Machine learning can discover previously unknown associations by exploring interaction patterns among variables in large-scale datasets [3]. In the global context, health data science—combining mathematics, statistics, computer science, and medicine—has quickly become an emerging and promising field to provide solutions to complex real-world health problems. 

However, inconsistent standards, insufficient interoperability, and limited quality of health data have hampered data sharing and integration. Technology optimism—which many people rely on to offer a vision of a brighter future—needs a second thought when considering healthcare practice. Social media should bring people together but can be misused to invade privacy and spread health misinformation and disinformation. Artificial intelligence (AI) may introduce bias and prejudice, which will exacerbate existing inequity in health and economic status. Digital devices provide tools for physicians but must overcome the suspicion on their validity and safety.

All of these examples remind us that when technical advances are integrated into a complex framework, such as a clinical practice or healthcare system, their success depends on many aspects. Advanced health technologies need rigorous evaluation for efficacy and safety before widespread adoption, and the ethical principles should be integrated into AI software engineering and design. It requires a better understanding of patients’ needs and healthcare values to harness the momentum of health data and technological innovation for affordable, accessible, and high-quality healthcare.

Given the health data science sphere’s interdisciplinary nature, communication and collaboration between healthcare professionals, policymakers, data scientists, ethicists, and engineers determine the success of data and innovation-driven transformative healthcare changes.

To generate high-quality evidence and serve as a platform for interdisciplinary discussion and debate on these challenging healthcare issues, we are launching this journal, a new Open Access Science Partner Journal. Our journal is committed to improving data and evidence-based decision-making, promoting the ethical application of cutting-edge technologies and analytic approaches, advancing the horizon of health data science, and creating value in real-world health practice.

This journal is aimed at closely working with the international academic community to maintain high-quality and ethical standards for research, translate the value of health data to benefit patients’ health and populations’ wellbeing, and achieve the journal’s mission—data for better health.
